# Soil Iron Content as a Predictor of Carbon and Nutrient Mobilization in Rewetted Fens

**DOI:** 10.1371/journal.pone.0153166

**Published:** 2016-04-06

**Authors:** Willem-Jan Emsens, Camiel J. S. Aggenbach, Ken Schoutens, Alfons J. P. Smolders, Dominik Zak, Rudy van Diggelen

**Affiliations:** 1 Ecosystem Management Research Group, Department of Biology, University of Antwerp, Universiteitsplein 1C, 2610 Wilrijk, Belgium; 2 KWR Watercycle Research Institute, P.O. Box 1072, 3430 BB Nieuwegein, The Netherlands; 3 Department of Aquatic Ecology and Environmental Biology, Institute for Wetland and Water Research, Radboud University Nijmegen, Heyendaalseweg 135 1, NL-6525 ED Nijmegen, The Netherlands; 4 B-WARE Research Centre, Toernooiveld 1, 6525 ED Nijmegen, the Netherlands; 5 Chemical Analytics and Biogeochemistry, Leibniz-Institute of Freshwater Ecology and Inland Fisheries, Müggelseedamm 301, D-12587 Berlin, Germany; 6 Department of Bioscience, Aarhus University, Vejlsøvej, 8600 Silkeborg, Denmark; West Chester University of Pennsylvania, UNITED STATES

## Abstract

Rewetted, previously drained fens often remain sources rather than sinks for carbon and nutrients. To date, it is poorly understood which soil characteristics stimulate carbon and nutrient mobilization upon rewetting. Here, we assess the hypothesis that a large pool of iron in the soil negatively affects fen restoration success, as flooding-induced iron reduction (Fe^3+^ to Fe^2+^) causes a disproportionate breakdown of organic matter that is coupled with a release of inorganic compounds. We collected intact soil cores in two iron-poor and two iron-rich drained fens, half of which were subjected to a rewetting treatment while the other half was kept drained. Prolonged drainage led to the mobilization of nitrate (NO_3_^-^, > 1 mmol L^-1^) in all cores, regardless of soil iron content. In the rewetted iron-rich cores, a sharp increase in pore water iron (Fe) concentrations correlated with concentrations of inorganic carbon (TIC, > 13 mmol L^-1^) and dissolved organic carbon (DOC, > 16 mmol L^-1^). Additionally, ammonium (NH_4_^+^) accumulated up to phytotoxic concentrations of 1 mmol L^-1^ in the pore water of the rewetted iron-rich cores. Disproportionate mobilization of Fe, TIC, DOC and NH_4_^+^ was absent in the rewetted iron-poor cores, indicating a strong interaction between waterlogging and iron-mediated breakdown of organic matter. Concentrations of dissolved phosphorus (P) rose slightly in all cores upon rewetting, but remained low throughout the experiment. Our results suggest that large pools of iron in the top soil of drained fens can hamper the restoration of the fen’s sink-service for ammonium and carbon upon rewetting. We argue that negative effects of iron should be most apparent in fens with fluctuating water levels, as temporary oxygenation allows frequent regeneration of Fe^3+^. We conclude that rewetting of iron-poor fens may be more feasible for restoration.

## Introduction

Widespread drainage is compromising the ability of the world’s groundwater-fed peatlands (“fens” hereinafter) to serve as sinks for nutrients and carbon [1,2]. In response to desiccation of organic soils, organic-bound nutrients are mineralized to inorganic mobile compounds [3,4], thereby contributing to pore and surface water eutrophication and loss of typical biodiversity. At the same time, rates of carbon dioxide emission increase upon drainage, which significantly impacts the world’s greenhouse gas budget [2]. Finally, rising concentrations of dissolved organic carbon (DOC) in surface waters adjacent to degraded peatlands have been related to the destabilization of carbon pools within the peat [5,6]. These high DOC loads have become subject to growing international concern as they cause ample environmental problems [7,8].

In response to the aforementioned trends, many countries are installing policies that aim to restore a significant portion of drained fens together with the vital ecosystem services that they provide. Fen restoration primarily focuses on the re-establishment of high groundwater levels, e.g. through ditch blockage. However, reports of excess mobilization of dissolved organic and inorganic carbon, ammonium (NH_4_^+^), and phosphate (PO_4_^3-^) into pore and surface water upon rewetting indicate that successful fen restoration is not always guaranteed at least in short-term [3,9]. The apparent difficulty in predicting restoration success by rewetting can be related to the complex and often diverse chemistry of fens: fens, much more than rainwater-fed bogs, vary greatly in cation, nutrient, electron acceptor and organic matter availability as well as pH and alkalinity. To date, all mechanisms and chemical characteristics that affect compound mobilization in drained peat soils are not well understood, which often results in an arbitrary or even random selection of fens that are listed as “suitable” for rewetting.

In this respect, past research has emphasized the importance of high concentrations of iron (Fe) in both soil and flooding water mainly because iron prevents mass mobilization of phosphate and reduced sulfur species into the pore water by providing sorption surfaces for both elements [10,11]. Based on those insights, the rewetting of iron-rich fens could be considered a relatively safe choice. However, potential negative effects of large iron pools in organic soils are usually neglected. In unpolluted fens and wetlands, iron (Fe^2+^-Fe^3+^) forms a dominant reduction-oxidation couple [12,13], and ferric iron often exceeds concentrations of other potential electron acceptors such as SO_4_^2-^ or NO_3_^-^. In fact, the “iron-redox wheel”, in which iron alternately shifts between the ferric (Fe^3+^) and ferrous (Fe^2+^) state in fluctuating redox environments, is potentially of major importance for carbon and nutrient cycling as labile organic matter is an electron donor in the microbe-mediated Fe-reduction reaction [13,14,15]. In a peatland that is influenced by Fe-inflow for example, an estimated 72% of anaerobic carbon mineralization is directly coupled with Fe^3+^ reduction, in contrast to only an estimated 7% in an upland bog that lacks Fe inflow [16]. In order for such iron-redox wheel to persist, fluctuating water levels are a prerequisite. In this respect, it is key to realize that human-induced rewetting rarely results in stable water levels year round: wet winters often result in temporarily flooded conditions while occasional long dry summers cause topsoil desiccation, even in “restored” fens [17,18]. This is partly related to irreversible shifts in the local hydrological system [18], as well as to physical alterations in the peat soil like the loss of the oscillation ability due to past drainage [19,20]. These physio-chemical alterations imply that rewetted fens may be relatively vulnerable to iron-induced decomposition.

Besides the direct relationship between iron reduction and decomposition, iron can additionally stimulate organic matter mineralization through indirect pathways. For example, iron promotes the production and activity of the extracellular enzyme phenol oxidase, which catalyzes the oxidation of decomposition-inhibiting phenolics [21,22]. Also, excess production of alkalinity coupled with Fe reduction raises soil buffering [23,24], thereby indirectly increasing rates of decomposition [25].

As past fen drainage has been shown to concentrate iron in the degraded top soil [23], the potential Fe-related soil reactivity in terms of carbon and nutrient mobilization in degraded fens could be substantially enhanced. The potential magnitude of such effect in natural peat soils has, however, never been documented. In this paper, we investigated if soil iron content is indeed a significant predictor of nutrient and carbon mobilization in drained fens upon rewetting. To test this hypothesis, we comparatively investigated the effects of experimental rewetting on four drained fens that vary in soil iron content.

## Materials and Methods

### Study areas and soil core collection

We selected four fens in the Pleistocene part of the Netherlands and Belgium that strongly differ in soil iron content: two fens (BM and LH) had relatively low- iron contents in soil (258 and 256 mmol kg^-1^ soil respectively), while two fens (ES and ZB) were considered iron-rich (537 and 1960 mmol kg^-1^ respectively, Table 1). All study sites were, historically, characterized by continuous upwelling of base-rich groundwater. Due to differences in geochemistry of the aquifers feeding the fens, the incoming groundwater of locations ES and ZB is relatively iron-enriched whereas the groundwater of BM and LH is relatively iron-poor, which explains the observed differences in total soil iron pools. Over the past century, land use intensification coupled with drainage has led to lowered groundwater levels and thus degradation of the top peaty soil layers at all sites (Von Post Humification Index upper 50 cm > 8). Current vegetation is characterized by a high presence of fen meadow species such as *Caltha palustris*, *Carex panicea*, *Cirsium palustre*, *Galium palustre*, *Juncus spp*., *Lotus uliginosus*, and *Holcus lanatus* in the herb layer and *Calliergon(ella) spp*., *Brachythecium spp*. and *Climacium dendroides* in the moss layer. To date, all sites are owned and protected by nature conservation agencies. We were granted permission to collect the peat cores from the following authorities: Staatsbosbeheer (sites BM and ES), Landschap Overijssel (site LH) and Natuurpunt (site ZB).

**Table 1:** Soil characteristics. Soil bulk density (kg L^-1^), organic matter content (%), Ammonium chloride-extractable P (NH_4_Cl-P), bicarbonate-dithionite extractable Fe and P (BD-Fe and BD-P), hydrochloric acid-extractable Fe, Al, Ca and P (HCl-Fe, HCl-Al, HCl-Ca, and HCl-P), and total Fe and P contents (in mmol kg^-1^) of the four study sites. Values (means ± SD) are based on samples (0–25 cm) collected in each of the 40 cores.

Parameter	Unit	Bennekomse Meent (BM)	Leijer Hooilanden (LH)	Elperstroom (ES)	Zwarte Beek (ZB)
Coordinates		52° 0'25.98"N; 5°35'48.80"E	52°38'32.71"N; 6°16'37.50"E	52°52'26.01"N; 6°39'32.96"E	51° 5'23.91"N; 5°19'10.69"E
Bulk density	kg L^-1^	0.13 ± 0.02	0.07 ± 0.01	0.15 ± 0.05	0.27 ± 0.04
OM-content	%	41.7 ± 7.9	81.8 ± 1.4	44.6 ± 13.3	22.8 ± 3.5
NH_4_Cl-P	mmol kg^-1^	0.018 ± 0.003	0.054 ± 0.012	0.031 ± 0.021	0.019 ± 0.007
BD-P	mmol kg^-1^	1.45 ± 0.55	2.72 ± 0.97	1.50 ± 0.43	1.55 ± 0.7
HCl-P	mmol kg^-1^	2.81 ± 0.54	11.16 ± 3.05	9.23 ± 3.54	50.64 ± 17.85
Total P	mmol kg^-1^	20.15 ± 3.6	44.85 ± 7.05	29.87 ± 12.81	144.8 ± 14.59
HCl-Al	mmol kg^-1^	113.9 ± 23.7	128.7 ± 14.5	157.4 ± 37.0	108.3 ± 18.6
HCl-Ca	mmol kg^-1^	126 ± 34.8	189.9 ± 37.3	99.1 ± 34.1	54.8 ± 11.6
BD-Fe	mmol kg^-1^	31.5 ± 11.8	55.5 ± 18.1	185.6 ± 84.5	294.7 ± 42.2
HCl-Fe	mmol kg^-1^	117.8 ± 29.1	174.4 ± 26.8	321.7 ± 112.7	778.1 ± 159.5
Total Fe	mmol kg^-1^	258 ± 62	256 ± 36	537 ± 203	1960 ± 331

At the time of soil core collection (early February 2014), groundwater levels averaged 10–20 cm below the fen surface. Within each fen, we chose a central area with a peaty soil layer of > 50 cm thickness. We then collected ten intact replicate soil cores of 45 cm x 12.5 cm (depth x diameter) in a 2x4m grid at each location. To extract a core, we manually forced hard-PVC tubes (diameter = 12.5 cm) of 50 cm length in the peat soil after carefully removing aboveground vegetation. As the PVC tubes were sharpened at the bottom, it was fairly easy to cut through the peat without causing compaction. Next, we dug a narrow hole alongside the PVC tube which allowed us to close the tube’s bottom with a PVC-lid before actually removing the tube. This allowed us to collect a relatively undisturbed soil core without losing pore water, which was crucial to minimize peat oxidation during transport. Directly after removal, the top of the PVC tube was sealed with cling film.

### Experimental design and sampling

**Fig 1:**
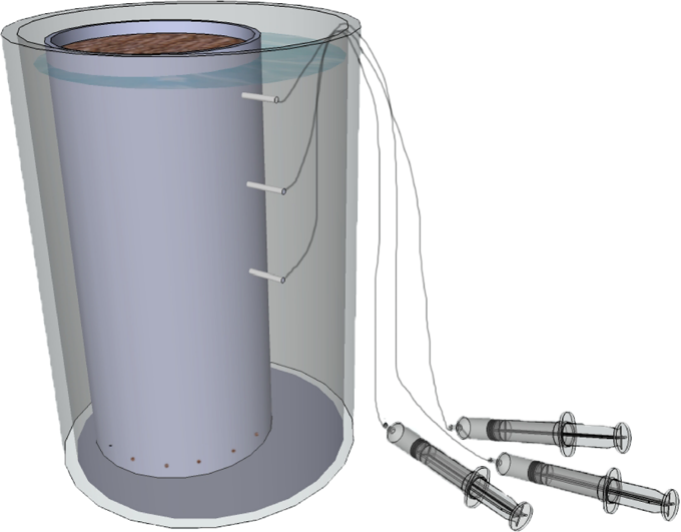
Experimental set-up. 40 intact vertical soil cores were collected in 4 drained fens using sharpened PVC tubes (45 x 12.5 cm), and were then placed in individual containers filled with stagnant de-oxygenized artificial groundwater. Tubes were perforated at the bottom to allow water inflow. Rhizons were placed at 5, 15 and 25 cm below the soil surface, and connected to vacuum-syringes. Half of the cores were rewetted to peat surface level, while the other half was kept moderately drained (water level 27 cm below peat surface level).

The 40 cores were placed in an unheated greenhouse where they were sheltered from direct sunlight. Average temperatures in the greenhouse ranged between 6.5 and 22.9°C (mean = 13.9 ± 3.8°C) over the course of the experiment, which ran from February through June 2014. We horizontally inserted 10 cm permanent Rhizon pore water samplers (pore size 0.2 μm, Rhizosphere Research Products, the Netherlands) at three different depths in each core: 5, 15 and 25 cm below the peat surface. Each Rhizon sampler was connected to a vacuum-syringe to allow the anaerobic extraction of pore water. Next, half of the soil cores received a rewetting treatment (water level at peat surface level), while the other half was drained (water level = 27 cm below the peat surface). As the water levels at the time of soil core collection averaged between 10–20 cm below the surface, the experimental water level manipulation not only triggered a rewetting effect but also a (more pronounced) drainage effect. Water level manipulation was accomplished by placing each PVC tube (perforated at the bottom) in a separate larger tube that was prefilled with the required amount of stagnant N_2_-deoxygenized artificial groundwater containing limited amounts of base minerals but no nutrients (i.e. N and P) or pollutants (i.e. S) (Fig 1). This in order to mimic rewetting by unpolluted, minerotrophic groundwater. Characteristics of the artificial groundwater were (means of five samples ± SE, in μmol L^-1^): pH = 7.0 ± 0.0, HCO_3_^-^ = 988 ± 73, Ca^2+^ = 608 ± 58, K^+^ = 18 ± 1, Na^+^ = 199 ± 41, Mg^2+^ = 5 ± 1, Cl^-^ = 145 ± 26. In order to allow gas exchange between peat soil and the surrounding atmosphere while preventing plant growth, we made three 3 mm holes in each top lid. This is sufficient for gas exchange, but hampers plant growth due to light limitation. Water levels were manually kept stable throughout the duration of the experiment. The design was full-factorial so that each combination of location (n = 4) x water level treatment (n = 2) was replicated five times. After initiation of the experiment, we first allowed the cores to stabilize for four days. On day four, we collected the first set of 120 pore water samples which we considered the starting point reference (t = 0 days). Next, pore water samples were collected biweekly and analyzed for pH and electrical conductivity (EC) using portable equipment (except t = 8 weeks). Pore water samples collected at t = 0, t = 30 and t = 127 were stored air-tight at 4°C and transported to the lab for further chemical analysis.

### Chemical analyses

Total inorganic carbon (TIC) was analyzed in the lab on an Infrared Gas Analyzer (ABB Advance Optima). Concentrations of NH_4_^+^ and NO_3_^-^ were determined colorimetrically on a continuous-flow Auto Analyzer system. Pore water subsamples were acidified by adding 0.7 ml 65% suprapure HNO_3_ per 100 ml sample and were analyzed with inductively coupled plasmaspectrometry (ICP, IRIS Intrepid II) for the following elements: Ca^2+^, Mg^2+^, K^+^, Na^+^, Fe_t_, Mn_t_, P_t_, S_t_, and Al_t_. Dissolved organic carbon (DOC) was analyzed using a Shimadzu TOC-VCPH Total Organic Carbon Analyser (Shimadzu Scientific Instruments, USA). Concentrations of CH_4_ and H_2_S were collected in the headspace of a 12 ml anaerobic glass vacuum tube that was prefilled with 0.5 ml 4% HCl, to which a subsample of 5 ml pore water was added. CH_4_ concentrations were measured with a Hewlett-Packard 5890 gas chromatograph (Avondale, California) equipped with a flame-ionization detector and a Porapak Q column (80/100 mesh) operated at 120°C with N^2^ as carrier gas. H_2_S concentrations were determined using a Packard 438A gas chromatograph equipped with a Carbopack B HT100 column (40/60 mesh) and a flame photometric detector.

Mineral saturation indices were calculated based on pore water measurements of pH and the following total concentrations of dissolved species: Ca, Mg, Fe, Mn, Na, K, Cl, Al, S, P, C, and N using PhreeqC [26].

Chemical characterization of the soils (Table 1) was done by sequential extractions on fresh soil to determine ammonium-chloride extractable P (NH_4_Cl-P) (= desorbable P), bicarbonate-dithionite extractable Fe and P (BD-Fe and BD-P (= reductant-soluble Fe and P)) and hydrochloric acid extractable Fe, Al, Ca, and P (HCl-Fe, HCl-Al, HCl-Ca, and HCl-P (= acid-soluble Fe, Al, Ca and P)) [27]. Fe, Al, and Ca concentrations of the chemical extracts (BD and/or HCl) were determined using inductively coupled plasma optical emission spectrometry (ICP-OES)I (Cap 6000 series, Thermo Fischer), and P concentrations of the chemical extracts were determined using the molybdenum blue method after acid digestion [27]. Precision and accuracy were better than 5% for Fe, Al, and Ca analysis and the detection limit was 2, 3 or 4 μM respectively. Total soil Fe and P was determined on ICP-OES after digesting 200 mg of oven-dried and ground soil with 4 mL HNO_3_ (65%) and 1 mL H_2_O_2_ (30%) using a microwave labstation (Milestone srl). Organic matter content (% dry weight) was determined by loss on ignition (4h at 550°C).

### Data analyses

We used Linear Mixed-Effect Modeling with REML estimation in SPSS (IBM SPSS Statistics 20) to assess the effects of soil iron content and water level on pore water concentrations of dissolved Fe, carbon (TIC, DOC and CH_4_) and macronutrients (NH_4_^+^, NO_3_^-^, total dissolved P). To this purpose, we made two groups based on soil iron content (treatment = “Iron content”): the two locations with mean soil iron content > 500 mmol kg^-1^ were classified as iron-rich sites (sites ES and ZB), and the remaining two locations with soil iron content < 500 mmol kg^-1^ as iron-poor sites (sites BM and LH). For each of these two groups, one half of the replicate cores was fully rewetted while the other half was drained (treatment = “Water level”). This setup resulted in a total of four different groups for data analysis. To correct for random between-site variation as well as for differences in means between sites, we added “Site ID (ES, ZB, BM or LH)” as a random factor nested within the treatment “iron content”. Although we collected data at different points in time, we did not add “Time” as an additional factor in the model for two reasons. First, visual data exploration reveals that the effect of “Time” is merely strengthening (i.e. differences between treatments become more pronounced as time progresses). Second, adding “Time” as a factor to the model would make the model unnecessarily complicated, as this would imply 3-way interactions between “Time”, “Iron content” and “Water level”. Hence, we simplify the Mixed Model by comparing treatment differences both at the start as well as at the end of the experiment (n = 0 and n = 127 days respectively) with tests for 2-way interactions between the fixed factors “Iron content” and “Water level”. We expect that the magnitude of the effect of rewetting (“Water level”) on carbon and nutrient mobilization depends on soil iron content, with higher carbon and nutrient mobilization in the rewetted iron-rich soil cores. For statistical analyses, values of the three pore water subsamples per soil core were averaged to attain column averages as well as to avoid pseudo-replication (S1 Table).

To further verify whether iron reduction is directly coupled with the mobilization of TIC, DOC and NH_4_^+^ after rewetting, we used Spearman’s rank correlation to compare the change in concentrations of total dissolved iron (ΔFe) from t = 0 days to t = 127 days with ΔTIC, ΔDOC and ΔNH_4_^+^. Correlations were conducted for the 20 rewetted cores and the 20 partly drained cores separately. Significance was accepted at the p < 0.05 level.

## Results

### pH and conductivity

**Fig 2:**
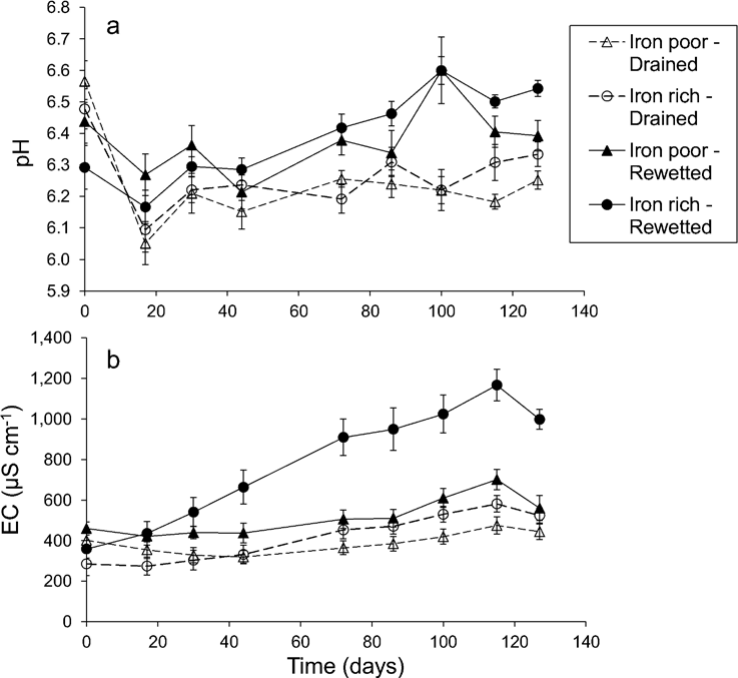
Changes in pore water pH and electrical conductivity (EC) in 40 soil cores. The cores differ in experimental water level treatment (rewetted or desiccated) and initial soil iron content (high or low). Soil cores were classified into 4 groups: rewetted iron-poor fens (n = 10 cores from 2 sites), desiccated iron-poor fens (n = 10 cores from 2 sites), rewetted iron-rich fens (n = 10 cores from 2 sites), and desiccated iron-rich fens (n = 10 cores from 2 sites). Dots represent group means ± SE.

Pore water pH in the peat columns remained relatively stable between 6.0 and 6.7 for all groups (Fig 2A). Over time, the rewetted soil cores gradually attained higher pH values than the drained soil cores. Highest final pH values were reached in the rewetted iron-rich cores, despite the fact that initial pH values at the start of the experiment were lowest in this group. Rewetting had a significant positive effect on pH values, but no interaction between water level and soil iron content was found (Table 2). Electrical conductivity (EC) in the partly drained soil cores as well as in the rewetted iron-poor soil cores remained between 200 and 600 μS cm^-1^ (Fig 2B). EC in the rewetted iron-rich soil cores however more than doubled over time, with the strongest increase during the first 70 days after rewetting. After 120 days, EC leveled around values of 1000 μS cm^-1^. We found a significant interaction effect between water level and iron content with significantly higher EC values in rewetted, iron-rich soil cores (Table 2).

**Table 2:** Output of the linear mixed-effect models. The models included two fixed factors “Water level (rewetted or drained)” and “Iron content (low or high)” and were corrected for the random factor “Site ID” (ZB, ES, BM or LH), with tests for interactions between soil iron content and water level. Dependent variables are mean pore water pH, EC, and concentrations of total dissolved iron (Fe), total inorganic carbon (TIC), dissolved organic carbon (DOC), methane gas (CH_4_), ammonium (NH_4_^+^), nitrate (NO_3_^-^) and total dissolved phosphorus (P) measured at the start (t = 0 days) and at the end of the experiment (t = 127 days).

Dependent variable	Fixed factor	0 days			127 days		
		df	F-value	P-value	df	F-value	P-value
pH (μmol L^-1^)	Water level	**1,34**	**6.65**	**0.014**	**1,34**	**28.92**	**<0.001**
	Iron content	1,2	0.71	0.488	1,2	2.21	0.276
	Water level * Iron content	1,34	0.23	0.633	1,34	1.10	0.301
EC (μS cm^-1^)	Water level	**1,34**	**8.00**	**0.008**	1,34	49.94	0.000
	Iron content	1,2	0.46	0.569	1,2	6.12	0.132
	Water level * Iron content	1,34	0.1	0.754	**1,34**	**18.42**	**<0.001**
Fe (μmol L^-1^)	Water level	1,34	0.94	0.339	1,34	58.80	0.000
	Iron content	1,2	0.13	0.752	1,2	20.68	0.045
	Water level * Iron content	1,34	0.00	0.979	**1,34**	**35.71**	**<0.001**
TIC (μmol L^-1^)	Water level	**1,34**	**6.84**	**0.013**	1,34	374.36	0.000
	Iron content	1,2	0.75	0.479	1,2	6.33	0.128
	Water level * Iron content	1,34	1.56	0.220	**1,34**	**34.53**	**<0.001**
DOC (μmol L^-1^)	Water level	1,34	0.31	0.580	1,34	96.52	0.000
	Iron content	1,2	0.01	0.943	1,2	1.48	0.347
	Water level * Iron content	1,34	0.26	0.612	**1,34**	**25.37**	**<0.001**
CH_4_ (μmol L^-1^)	Water level	1,34	0.48	0.494	**1,34**	**6.61**	**0.014**
	Iron content	**1,2**	**127.78**	**<0.001**	1,2	0.03	0.872
	Water level * Iron content	1,34	1.22	0.276	1,34	0.02	0.889
NH_4_^+^ (μmol L^-1^)	Water level	1,34	1.45	0.237	1,34	69.69	0.000
	Iron content	1,2	3.26	0.213	1,2	5.68	0.140
	Water level * Iron content	1,34	1.00	0.324	**1,34**	**29.33**	**<0.001**
NO_3_^-^ (μmol L^-1^)	Water level	1,34	7.47	0.010	**1,34**	**314.32**	**<0.001**
	Iron content	1,2	8.00	0.106	1,2	0.11	0.772
	Water level * Iron content	**1,34**	**9.04**	**0.005**	1,34	0.29	0.591
P (μmol L^-1^)	Water level	1,34	0.30	0.587	**1,34**	**40.98**	**<0.001**
	Iron content	1,2	1.40	0.359	1,2	0.96	0.430
	Water level * Iron content	1,34	0.23	0.632	1,34	0.98	0.329

### Carbon and nutrient mobilization

**Fig 3:**
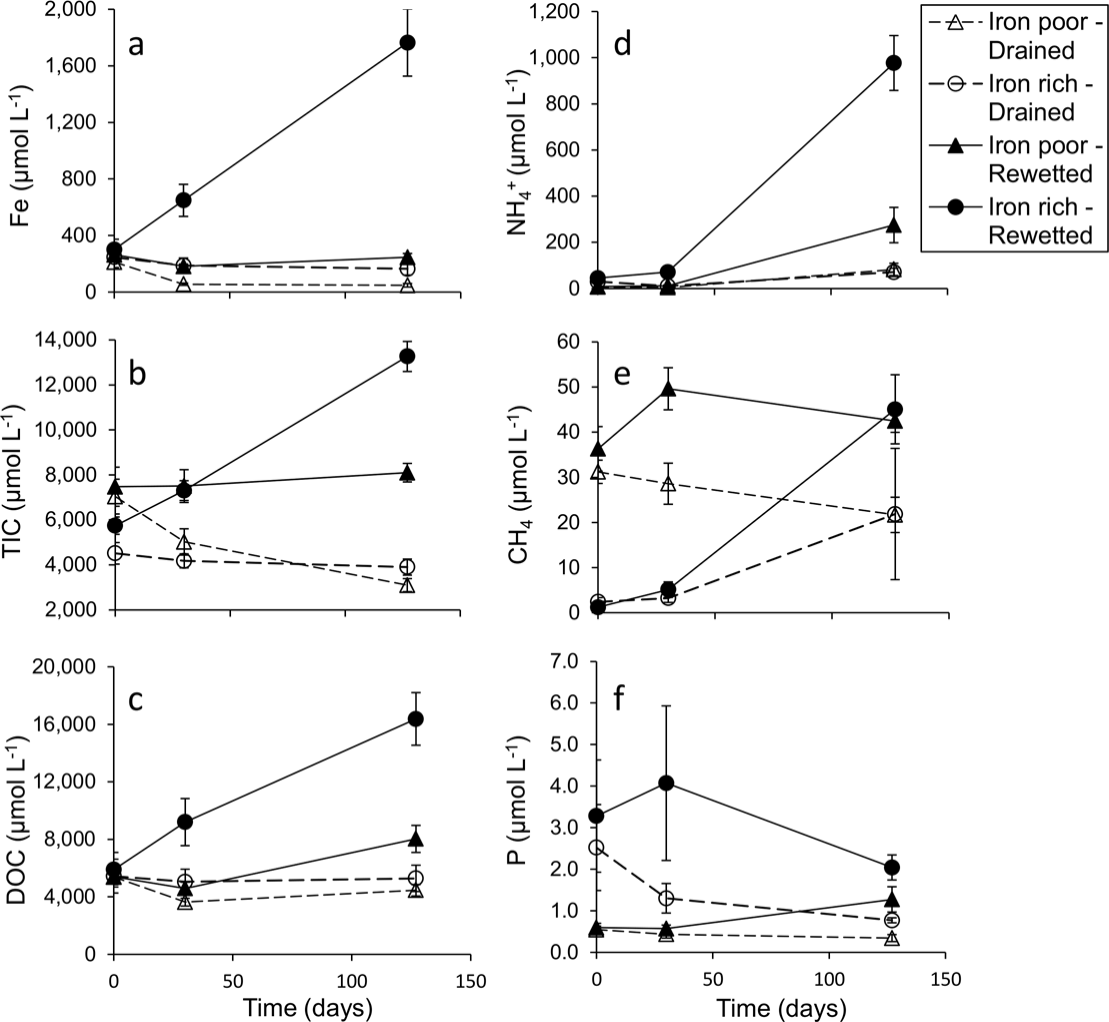
Iron, nutrient and carbon mobilization. Mobilization of (a) dissolved iron, (b) total inorganic carbon, (c) dissolved organic carbon, (d) ammonium, (e) methane and (f) total dissolved phosphorus over time (t = 0, 30 and 127 days) in the pore water of 40 soil cores that differ in experimental water level treatment (rewetted or desiccated) and initial soil iron content (high or low). Soil cores were classified into 4 groups: rewetted iron-poor fens (n = 10 cores from 2 sites), drained iron-poor fens (n = 10 cores from 2 sites), rewetted iron-rich fens (n = 10 cores from 2 sites), and drained iron-rich fens (n = 10 cores from 2 sites). Dots represent group means ± SE.

**Fig 4:**
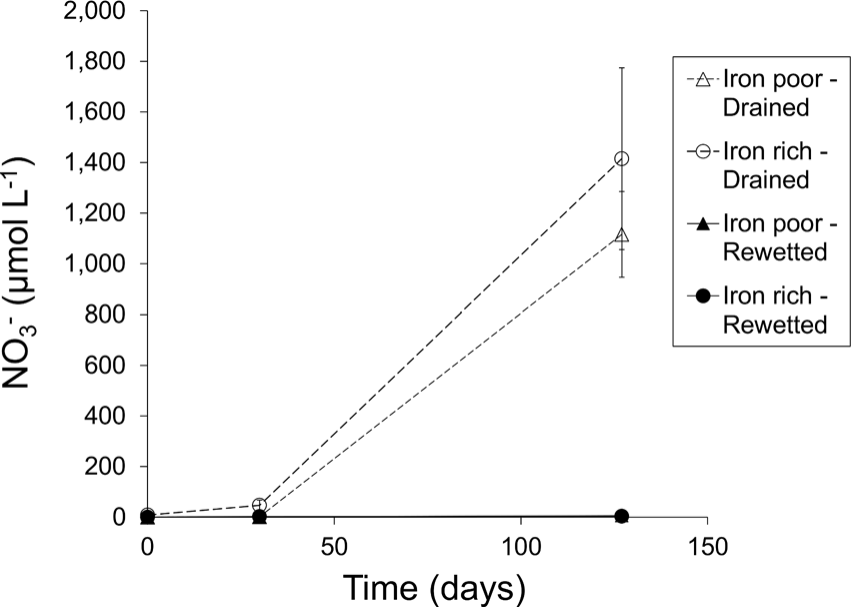
Nitrate mobilization. Mobilization of nitrate (NO_3_^-^) over time (t = 0, 30 and 127 days) in the pore water of 40 soil cores that differ in experimental water level treatment (rewetted or drained) and initial soil iron content (high or low). Soil cores were classified into 4 groups: rewetted iron-poor fens (n = 10 cores from 2 sites), drained iron-poor fens (n = 10 cores from 2 sites), rewetted iron-rich fens (n = 10 cores from 2 sites), and drained iron-rich fens (n = 10 cores from 2 sites). Dots represent group means ± SE.

At the start of the experiment (t = 0 days) the factors “water level” and “iron content” had no significant effect on pore water concentrations of dissolved Fe, DOC, NH_4_^+^ and P (Table 2, Fig 3), indicating that hydrochemical conditions in all four groups were relatively similar. In the iron-poor soil cores, initial concentrations of CH_4_ were higher_,_ while there was an interaction effect of water level with iron content for concentrations of NO_3_^-^ (Table 2, Fig 4), with slightly higher NO_3_^-^ concentrations in the drained Fe-rich cores (in the order of a few μmolars). Initial concentrations of TIC were slightly higher in all rewetted cores.

At the end of the experiment (t = 127 days), the drained soil cores had responded to desiccation in a similar manner regardless of soil iron content (Fig 3). Prolonged drainage (> 30 days) led to mass mobilization of NO_3_^-^ (> 1 mmol L^-1^) at all locations (Fig 4), and this effect was independent of soil iron content (Table 2).

Experimental rewetting led to a significant increase in pore water concentrations of Fe, TIC, DOC, NH_4_^+^, P and CH_4_ (Table 2, Fig 3). However, hydrochemical conditions in the rewetted iron-rich soil cores differed markedly from the conditions in the rewetted iron-poor soil cores (Table 2, Fig 3). For the variables Fe, TIC, DOC, and NH_4_^+^, we found strong positive interactions between water regime and soil iron content: concentrations of Fe, TIC, DOC and NH_4_^+^ increased in response to rewetting, but the magnitude of this effect was much stronger in the iron-rich soil cores. Concentrations of both Fe and NH_4_^+^ reached more than 1 mmol L^-1^ in the rewetted iron-rich soil cores, while the accumulation of TIC and DOC had almost doubled compared to the rewetted iron-poor soil cores, reaching final mean concentrations of >13 mmol L^-1^ and >15 mmol L^-1^ respectively. Final CH_4_ concentrations did not differ between iron-rich and iron-poor soil cores and had increased after rewetting, but we observed a delay in CH_4_ accumulation in the rewetted iron-rich soil cores (Fig 3E). Pore water concentrations of total dissolved P increased slightly after rewetting, but in the iron-rich soil cores an initial increase to concentrations of 4.0 μmol L^-1^ was followed by a gradual decrease to concentrations of 2 μmol L^-1^ (Fig 3F). Initial mean (t = 0 days) concentrations of total dissolved sulfur (S_t_) were low in all treatments (< 100 μmol L^-1^, S1 Fig), while concentrations of sulfide remained below detection limit (< 0.1 μmol L^-1^) throughout the course of the experiment (results not shown).

**Fig 5:**
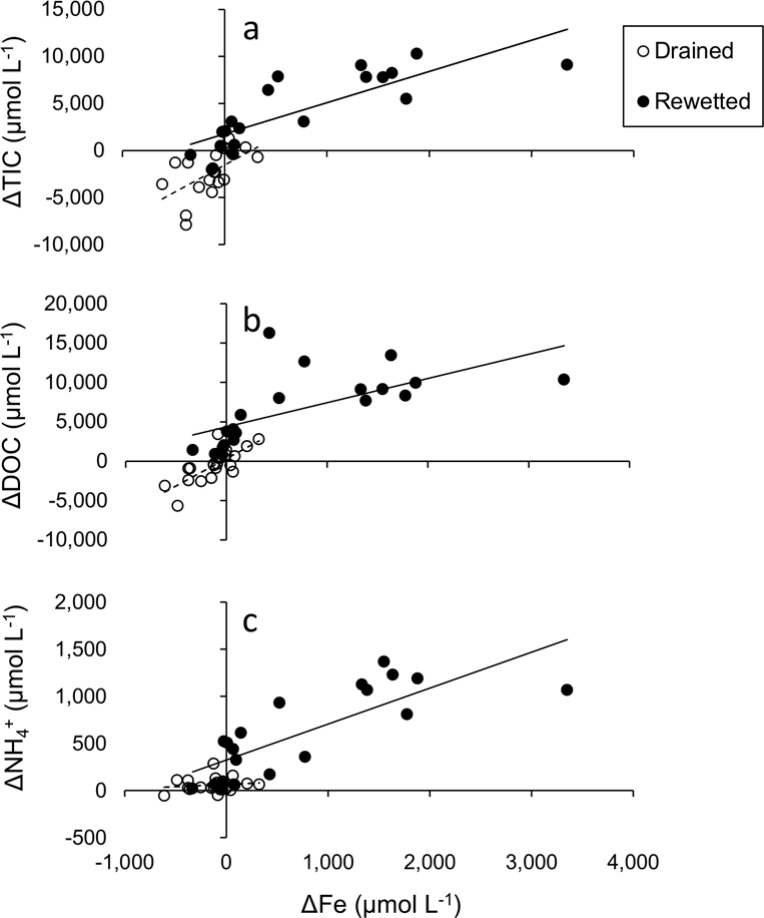
Relationship between iron, TIC, DOC and NH4+. Correlations between the change in pore water Fe concentrations (ΔFe) and the change in concentrations of (a) total inorganic carbon (ΔTIC), (b) dissolved organic carbon (ΔDOC) and (c) ammonium (ΔNH_4_^+^) (in **μ**mol L^-1^) in 20 rewetted and 20 drained soil cores over 127 days (n = 4 sites).

In addition, the change in concentrations of dissolved Fe throughout the experiment in the rewetted cores (ΔFe) correlated positively with ΔTIC (rho = 0.86, df = 18, p < 0.001, Fig 5A), ΔDOC (rho = 0.85, df = 18, p < 0.001, Fig 5B), and ΔNH_4_^+^ (rho = 0.82, df = 18, p < 0.001, Fig 5C). In the drained soil cores ΔFe also correlated with ΔTIC (rho = 0.69, df = 18, p < 0.001, Fig 5A) and ΔDOC (rho = 0.78, df = 18, p<0.001, Fig 5B), but not with ΔNH_4_^+^ (rho = 0.08, df = 18, p = 0.7, Fig 5C).

### Mineral saturation indices

After 127 days, saturation for siderite (FeCO_3_) was found in all of the rewetted soil cores, with supersaturation in the iron-rich cores. Supersaturation for rhodochrosite (MnCO_3_) was found only in a subset of rewetted iron-rich cores, but not in the iron-poor cores. Vivianite (Fe(II)_3_(PO_4_)_2_.8H_2_O) precipitation was predicted in some (but not all) of the rewetted iron-rich soil cores. Slight supersaturation for calcite (CaCO_3_) was sporadically found in the cores with highest pH values (> 6.4), which only occurred in some alkaline layers of the rewetted iron-rich cores of site ES. Ca-P precipitation as hydroxyapatite (Ca_5_(PO_4_)_3_(OH)) was unlikely due to strong undersaturation.

## Discussion

Rewetting of drained organic soils unambiguously triggered the mobilization and accumulation of NH_4_^+^, TIC and DOC into the pore water, but this rewetting effect was disproportionately stronger in fens with large iron pools, suggesting a strong iron-mediated breakdown of organic matter [14,15]. Concentrations of dissolved P however only rose slightly, and, in the iron-rich cores, had again dropped to low levels at the end of the experiment, indicating a P sink.

### Nutrient (P, NO_3_^-^ and NH_4_^+^) mobilization

Soil desiccation increases mineralization rates of organic matter. In response, organic-bound nutrients are converted into inorganic mobile ions, which is why drainage of wet soils is coupled with eutrophication [4]. In our dataset, this eutrophication effect corresponds with the observed increase in concentrations of NO_3_^-^ up to > 1 mmol L^-1^. Conversely, accumulation of NO_3_^-^ did not occur in the rewetted soil cores, as no nitrification of ammonium can take place under anaerobic conditions. However, although rewetting can be an effective mechanism to prevent aerobic decomposition and accumulation of nitrate in organic soils, rewetting does not necessarily lower nutrient availability in general. Compared to the drained soil cores, we measured slightly higher concentrations of dissolved P and much higher concentrations of ammonium in the rewetted cores. For P, this apparent discrepancy is directly related to the differences in redox-state between both water level treatments. Higher concentrations of dissolved P in the rewetted cores can be linked to anaerobic reduction processes in which organic matter and organic-P are mineralized, as well as to the well-known redox-sensitive dissolution of P from amorphous Fe-(hydr)oxides under anoxic conditions [28,29,30]. The latter mechanism is particularly relevant in drained and degraded groundwater-fed fens, in which a large part of the inorganic P pool is already iron-bound [3,23]. Although we had expected a mass release of P in the rewetted iron-poor soils in particular, pore water P concentrations only rose to low concentrations of 1.3 μmol L^-1^. In the rewetted iron-rich cores, an initial increase in total dissolved P during the first (t = 30 d) phase of rewetting was soon followed by a decrease in concentrations of dissolved P to 2 μmol L^-1^. Computed supersaturation for vivianite in some of the rewetted iron-rich cores at the end of the experiment suggests vivianite precipitation under anoxic iron-rich conditions, which can be an effective sink for soluble P but is generally slow to obtain equilibrium [31,32,33]. Additionally, other phases than iron compounds may have controlled the solubility of P in the pore water after rewetting, as P can be resorbed to redox-insensitive compounds such as aluminium oxides [34,35]. It remains unclear to what extent this occurred in our study. Also the role of Ca is not clear, however apatite formation seems to be negligible in our sites. Finally, it should be noted that we measured total P in the pore water and not inorganic P. Therefore, we cannot exclude that part of the P is bound to DOM. Since inorganic P is denoted usually as “bioavailable” we might overestimate the amount of P which is mostly relevant for eutrophication. Concentrations of inorganic P may thus be even lower than the results reported here. These results suggest that pore water P mobilization upon fen rewetting can remain well below eutrophic thresholds, which is in contrast with most studies on fen rewetting [30,36]. We relate this primarily to the low S-loads in our study sites, as sulfates and sulfides enhance internal eutrophication [25].

In contrast to P, NH_4_^+^ concentrations rose to potentially phytotoxic levels (1 mmol L^-1^) in the iron-rich rewetted cores, while remaining considerably lower in the rewetted iron-poor cores (< 0.3 mmol L^-1^). As inflow and outflow of ammonium as well as plant uptake was absent in our closed experiment, only in situ NH_4_^+^ production/mobilization and transformation/adsorption plays a role. At least two nonexclusive processes must be taken into account in order to explain the observed interaction effect between rewetting and soil iron content on ammonium accumulation. First, a disproportionately high accumulation of NH_4_^+^ in the iron-rich soil cores can be related to the iron-mediated anaerobic breakdown of organic matter, which triggers the conversion of organic-N to inorganic-N and releases NH_4_^+^ into the pore water. Here, reduced nitrification rates prevent NH_4_^+^ transformation [37]. The positive correlations between pore water accumulation of Fe and NH_4_^+^ as well as Fe and TIC in our dataset indeed suggest such iron-mediated breakdown of organic matter coupled with NH_4_^+^ release, as all compounds are products of anaerobic decomposition processes. Second, we explored the possibility that a larger quantity of mineral NH_4_^+^ was, by chance, already adsorbed to the adsorption complex of the two iron-rich fens. Rewetting with artificial groundwater increases competition for soil exchange sites and may stimulate ammonium release from the soil [38]. However, previously collected field data on KCl-extractable NH_4_^+^ at all four study sites indicates that the amount of NH_4_^+^ that can be released from the adsorption complex of one litre of soil equals an estimated 0.98 and 0.97 mmol in the iron-poor sites (site BM and LH respectively) and 1.07 and 0.54 mmol in the iron-rich sites (site ES and ZB respectively). Therefore, the differential response of iron-rich and iron-poor fens is unlikely to be explained by desorption. Finally, nitrate reduction is another well-known mechanism for NH_4_^+^ production [39], and this reaction can be mediated by iron compounds [40]. However, this pathway is unlikely to be important in our experiment as this would require a large pool of nitrate. In our study, initial pore water NO_3_^-^ concentrations in the peat cores were only in the order of a few μmolars, while field data on KCl-extractable NO_3_^-^ suggests values close to or even below detection limit at all study sites.

### Carbon (DOC, TIC and CH_4_) production

Throughout the experiment, mean pore water DOC concentrations in the drained and rewetted iron-poor soil cores remained within the range of 3.5–9.1 mmol L^-1^, with slightly elevated concentrations in the latter. In the rewetted iron-rich soil cores however, DOC accumulation reached high mean levels of 16 mmol L^-1^ (> 192 mg L^-1^). Such link between iron concentrations and disproportionate DOC mobilization has also been observed in the field [21,41]. We see two nonexclusive reasons for the positive correlation between DOC mobilization and soil iron content in the rewetted soil cores. First, the same mechanism that explains NH_4_^+^-mobilization affects DOC mobilization as well, i.e. the iron-mediated decomposition of organic matter. As DOC is a product of decomposition [42], increased rates of litter decomposition (either directly or indirectly) coupled with a large pool of soil iron should stimulate DOC release into the pore water. Second, large quantities of DOM can be adsorbed to Fe(III)(hydro)xides under oxic conditions [43,44], so that Fe(III)-reduction triggers the dissolution of Fe-DOC coagulates. As pointed out in recent research [44], such Fe-DOC coagulation in oxic soil layers serves as a barrier to DOC efflux from semi-terrestrial environments, but such barrier disappears upon rewetting. Although the quantitative contribution of each process to DOC accumulation in rewetted Fe-rich soils cannot be disentangled in our experiment, it is nonetheless clear that large-scale rewetting of drained iron-rich fens (in contrast to iron-poor fens) triggers a strong mobilization of DOC, thereby increasing DOC fluxes towards adjacent water catchments.

In non-calcareous fens (including our study sites), high concentrations of TIC are mainly related to in-situ anaerobic reduction processes coupled with organic matter decomposition [25]. Upon rewetting, hydrogen ions are consumed during anaerobic decomposition while HCO_3_^-^ and CO_2_ (TIC) are produced, and waterlogged conditions prevent rapid CO_2_-degassing towards the atmosphere. In our experiment, excess accumulation of TIC and a sharp rise in pH in the rewetted iron-rich cores, but to a lesser extent in the rewetted iron-poor cores, again points towards iron-induced anaerobic decomposition of organic matter in rewetted soils. For degraded iron-rich fens in particular, a considerable part of TIC production is indeed directly coupled with iron reduction [23]. Likewise, the reduction of nitrates or sulfates also produces TIC and alkalinity [25,45], but given the low mean pore water concentrations of these parameters at the start of the experiment (< 9 μmol L^-1^ and < 100 μmol L^-1^ respectively), this is unlikely to explain excess TIC production in our soil cores. It should be noted however that we only measured carbon accumulation in the pore water, but not total carbon fluxes towards the atmosphere. As oxygen is the most favorable electron acceptor, total carbon emissions (in the form of CO_2_) are most likely highest in all of the drained cores. Here, the generally rapid degassing of CO_2_ in aerated soils prevents CO_2_ accumulation in the pore water, so that flux measurements would be needed to estimate actual C loss to the atmosphere.

Organic soils that have been subject to prolonged desiccation generally require a longer time lag before significant CH_4_ accumulation, as electron acceptors have had sufficient time to regenerate [46]. Although methanogenesis can occur on a micro-scale despite the presence of energetically favorable electron acceptors, significant CH_4_ production is expected to only take place after sequential consumption of these electron acceptors by micro-organisms [21,47]. In our dataset, we observe a clear (an estimated ± 50–100 days) delay in methane accumulation in the rewetted iron-rich cores but not in the iron-poor cores, which, again, suggests that ferric iron is being reduced as an energetically favorable electron acceptor.

### Management implications

In the past, it has been emphasized that high concentrations of iron in soil and flooding water are beneficial for the restoration of wet ecosystems as iron prevents mobilization of phosphates and sulfides into pore and surface water [10,11,30]. This is particularly relevant in heavily degraded P-eutrophied or S-polluted wetlands that are characterized by very low soil Fe:S and Fe:P ratios [3,25]. In unpolluted mesotrophic fens however, the P- and S-binding service of iron may be of much less importance for the vitality of the fen system. In our experiment for example, concentrations of sulfide remained below detection limit both in iron-rich and iron-poor fens, while concentrations of dissolved P never reached eutrophic thresholds. Under such conditions, positive effects of iron may be overshadowed by negative effects. In this respect, many restored fens are, despite rewetting, characterized by seasonal fluctuations in water levels (and thus redox conditions [17,18]), and large pools of accumulated iron in the top soil [23]. Under such conditions, the iron redox cycle in which iron alternately shifts between the ferric (Fe^3+^) and ferrous (Fe^2+^) state can cause a continuous positive feedback on organic matter mineralization coupled with nutrient and carbon mobilization [14]. Here, repeated mass mobilization of TIC, DOC and NH_4_^+^ in each rewetting cycle can be expected to impact the functioning of the fen ecosystem and downstream systems. Although our data do not allow for an accurate prediction of how much carbon is released or mineralized with each oxidation/rewetting cycle, we can make a rough estimate based on several assumptions. We assume that a major part of DOC and TIC in soil water is the product of organic matter decomposition and associated processes like iron reduction. According to a simplified calculation (e.g. disregarding CO_2_ emissions towards the atmosphere) about 0.8% of total carbon in iron-rich cores and 0.17% of total carbon in iron-poor cores must be dissolved to reach the final mean sum of DOC and TIC concentrations of 18 mM or 3.5 mM (**Δ**TIC + **Δ**DOC), respectively. This estimate is based on i) different organic matter content and dry bulk density for iron-rich soils (means = 0.326 kg:kg, 0.21 kg L^-1^) vs. iron-poor soils (means = 0.617 kg:kg, 0.1 kg L^-1^) (Table 1), ii) the assumption that one litre of water corresponds roughly with one litre of soil, and iii) that organic matter has an average C fraction of 0.4 (kg:kg). Accordingly, one litre of soil of the iron-rich fens holds on average 27 g C and of the iron-poor fens holds on average 25 g C compared to **Δ**DOC+**Δ**TIC concentrations of 216 mg C L^-1^ (18 mM) and 42 mg C L^-1^ (3.5 mM) in pore water of iron-rich cores vs. in iron-poor cores which equates to percentage details given above. Therefore, if the goal of a fen rewetting project is to restore a low-productive fen that provides vital ecosystem services such as long-term carbon sequestration and nutrient retention, we suggest that the restoration of unpolluted drained iron-poor fens should deserve priority over the restoration of drained iron-rich fens.

### Conclusions

The biogeochemical effects of rewetting on drained fens are often unpredictable and site-dependent, and many rewetted fens remain sources rather than sinks for carbon and nutrients. In our comparative study, soil cores collected in drained iron-rich fens were characterized by a disproportionate mobilization of Fe, TIC, DOC and NH_4_^+^ in the pore water after rewetting, while such disproportionate mobilization after rewetting was absent in soil cores collected in drained iron-poor fens. Concentrations of dissolved P remained well below eutrophic thresholds, indicating a P sink even in rewetted fens. Our results suggest that high iron pools in organic soils interact with water regime, and rewetting stimulates strong iron-mediated organic matter mineralization coupled with carbon and inorganic nutrient (NH_4_^+^) mobilization. We conclude that fen restoration by rewetting may be more effective in (unpolluted) iron-poor fens.

## Supporting Information

S1 Fig Sulfur mobilization. Mobilization of total dissolved Sulfur (S) over time (t = 0, 30 and 127 days) in the pore water of 40 soil cores that differ in experimental water level treatment (rewetted or drained) and initial soil iron content (high or low). Soil cores were classified into 4 groups: rewetted iron-poor fens (n = 10 cores from 2 sites), drained iron-poor fens (n = 10 cores from 2 sites), rewetted iron-rich fens (n = 10 cores from 2 sites), and drained iron-rich fens (n = 10 cores from 2 sites). Dots represent group means ± SE.
(TIFF)

S1 Table Dataset with raw data. (XLSX)
