# Supplementary figures and images for: Soil Iron Content as a Predictor of Carbon and Nutrient Mobilization in Rewetted Fens
Source: PLoS One. 2016 Apr 6;11(4):e0153166. doi: 10.1371/journal.pone.0153166 (PMC4822970; doi:10.1371/journal.pone.0153166)

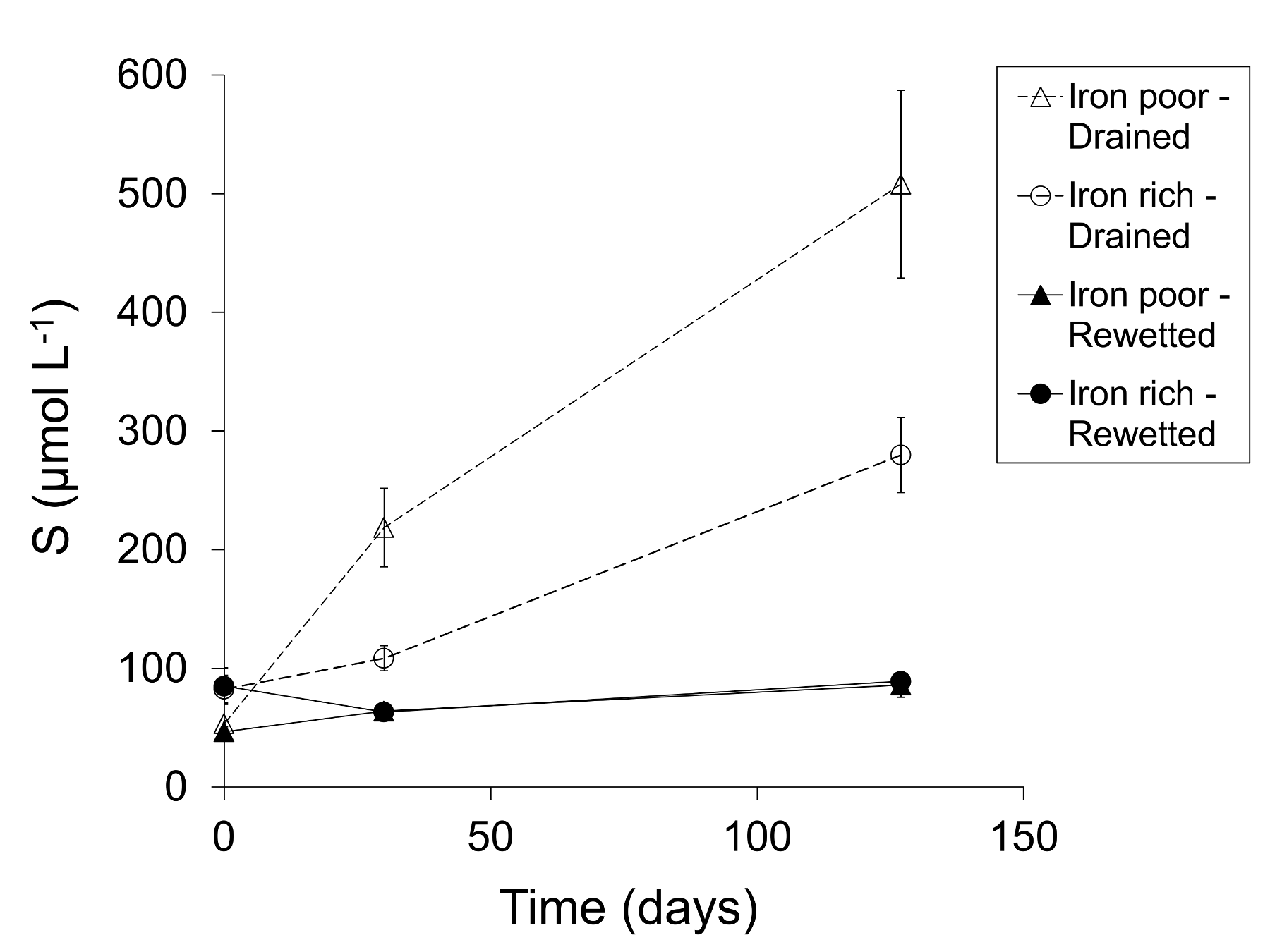

Supplement: S1 Fig — Mobilization of total dissolved Sulfur (S) over time (t = 0, 30 and 127 days) in the pore water of 40 soil cores that differ in experimental water level treatment (rewetted or drained) and initial soil iron content (high or low). Soil cores were classified into 4 groups: rewetted iron-poor fens (n = 10 cores from 2 sites), drained iron-poor fens (n = 10 cores from 2 sites), rewetted iron-rich fens (n = 10 cores from 2 sites), and drained iron-rich fens (n = 10 cores from 2 sites). Dots represent group means ± SE. (TIFF) [file pone.0153166.s001.tiff]
